# Bioactive Potential of Minor Italian Olive Genotypes from Apulia, Sardinia and Abruzzo

**DOI:** 10.3390/foods10061371

**Published:** 2021-06-14

**Authors:** Wilma Sabetta, Isabella Mascio, Giacomo Squeo, Susanna Gadaleta, Federica Flamminii, Paola Conte, Carla Daniela Di Mattia, Antonio Piga, Francesco Caponio, Cinzia Montemurro

**Affiliations:** 1 Institute of Biosciences and BioResources, National Research Council (IBBR-CNR), Via Amendola 165/A, 70125 Bari, Italy; 2 Spin off Sinagri s.r.l., University of Bari Aldo Moro, Via Amendola 165/A, 70125 Bari, Italy; cinzia.montemurro@uniba.it; 3 Department of Soil, Plant and Food Sciences, University of Bari Aldo Moro, Via Amendola 165/A, 70125 Bari, Italy; mascioisa@gmail.com (I.M.); giacomo.squeo@uniba.it (G.S.); sanna14@hotmail.it (S.G.); francesco.caponio@uniba.it (F.C.); 4 Faculty of Bioscience and Technology for Agriculture, Food and Environment, University of Teramo, Via Renato Balzarini 1, 64100 Teramo, Italy; fflamminii@unite.it (F.F.); cdimattia@unite.it (C.D.D.M.); 5 Dipartimento di Agraria, University of Sassari, Viale Italia 39/A, 07100 Sassari, Italy; pconte@uniss.it (P.C.); pigaa@uniss.it (A.P.); 6 Institute for Sustainable Plant Protection–Support Unit Bari, National Research Council (IPSP-CNR), Via Amendola 165/A, 70125 Bari, Italy

**Keywords:** *Olea europaea*, autochthonous cultivars, molecular fingerprinting, polyphenol content, gene expression, fruit developmental stages

## Abstract

This research focuses on the exploration, recovery and valorization of some minor Italian olive cultivars, about which little information is currently available. Autochthonous and unexplored germplasm has the potential to face unforeseen changes and thus to improve the sustainability of the whole olive system. A pattern of nine minor genotypes cultivated in three Italian regions has been molecularly fingerprinted with 12 nuclear microsatellites (SSRs), that were able to unequivocally identify all genotypes. Moreover, some of the principal phenolic compounds were determined and quantified in monovarietal oils and the expression levels of related genes were also investigated at different fruit developmental stages. Genotypes differed to the greatest extent in the content of oleacein (3,4-DHPEA-EDA) and total phenols. Thereby, minor local genotypes, characterized by stable production and resilience in a low-input agro-system, can provide a remarkable contribution to the improvement of the Italian olive production chain and can become very profitable from a socio-economic point of view.

## 1. Introduction

Olive (*Olea europaea* ssp. *europaea*) is considered among the historically and traditionally most important crops in the world and especially in the Mediterranean basin, where it has been cultivated for centuries not only for nutrition but also for cultural and religious reasons. In particular, the strategic geographical position of Italy in a temperate area has favored olive cultivation and therefore the enrichment of its germplasm over time, which is estimated to include about 800 cultivars [1,2]. The great diffusion of olive trees in the Italian territory highlights its importance for production, economy and local traditions, as proven and documented by innumerable historical catalogues and archives up to the XIII century B.C. [3].

As a consequence of the general awareness about the loss of plant genetic diversity and the drastic climate change currently underway, the attention of the scientific community has been recently put toward more sustainable agriculture, as opposed to the intensive, mono-cultivar farming systems, and to the safeguard of plant biodiversity as source of new interesting traits. Similarly to other cultivations, also for olive, a valid and intriguing opportunity to help in overcoming these issues is offered by the unexploited or still poorly characterized germplasm, including local or minor genotypes, i.e., autochthonous landraces generally spread at regional level and well adapted to specific pedoclimatic conditions in traditional groves with very low agronomic input [4]. These genotypes could represent an interesting reservoir of useful traits, such as nutraceutical and antioxidant compounds and/or resistances to environmental stresses, that makes their diffusion in the market and their utilization in breeding programs extremely promising. In this perspective, the first step for the valorization of these genotypes is represented by their fine characterization that provides the genetic identity of both plant material and derived products (drupes and oils) and that guarantees an unequivocal varietal recognition. Microsatellite markers (SSRs) are among the most simple, fast and economic molecular tools widely used for olive varietal identification [5,6,7,8] and for food tracking and tracing [9,10,11,12,13].

The extra virgin olive oil is considered a fundamental element of the Mediterranean diet [14], thus representing one of the pillars of the Italian economy. Being the most important fat source in the human diet, olive oil is characterized by a distinctive high content of mono-unsaturated fatty acids (MUFA) and by a certain level of secondary metabolites with nutraceutical properties, such as polyphenols [15]. The most abundant polyphenol class in olive oil is represented by the secoiridoids, i.e., complex molecules including oleuropein, ligstroside and their derivates (oleacein, oleocanthal, an isomer of oleuropein aglycone called 3,4-DHPEA-EA and the ligstroside aglycone also known as *p*-HPEA-EA), the latter compounds being produced during olive oil mechanical extraction. Polyphenols also include two important phenolic alcohols worthy to be mentioned for their antioxidant power, i.e., tyrosol and hydroxy-tyrosol [16].

A huge literature has been dedicated to the healthy value of polyphenols [17,18,19,20]. Numerous research has demonstrated their positive effects in contrasting cardiovascular diseases thanks to their anti-inflammatory and -oxidative actions [21,22,23,24]. In particular, polyphenols are able to bind low-density lipoprotein (LDL), avoiding its oxidation [25], modulate angiogenic responses [26], protect against endothelial dysfunction [27] and contribute to decrease the blood pressure [28]. Moreover, the positive effect of tyrosol and hydroxy-tyrosol against reactive oxygen species has been deeply investigated [29,30,31,32].

Furthermore, some phenolic compounds, especially oleuropein, ligstroside aglycone and derived molecules, are responsible for the pungency and bitter taste of olive oil [33,34,35,36]. For this reason, in recent decades, breeders and researchers focused on polyphenols as new trait of interest to satisfy the increasing request of high-quality olive oil for nutritional and organoleptic aspects [37,38,39].

The biosynthetic pathway of olive secoiridoids has been studied and proposed by several authors, even though it is not completely clarified yet. Transcriptomic and metabolomic analysis of developing olive fruits and leaves in different cultivars have shed light on the enzymes involved in polyphenol metabolism [40,41,42,43,44].

The main aim of this work was the valorization of some minor olive genotypes and cultivars, autochthonous of Central and Southern Italy, that are still poorly studied, but interesting for both their resilience and adaptation to low-input agriculture and for the quality of olive oil. The application of SSR markers has been necessary to obtain a clear molecular fingerprinting of these genotypes, as a recognized tool of primary importance for certifying plant material production, varietal tracing and authenticity testing. Moreover, an integrated approach of oil biochemical characterization, with particular focus on tyrosol, hydroxy-tyrosol and oleacein, coupled with a genetic expression study of key enzymes involved in the first steps of their biosynthesis, has contributed to clarify the molecular mechanisms underlying the olive polyphenol biosynthetic pathway and to further valorize the Italian olive germplasm.

## 2. Materials and Methods

### 2.1. Plant Material and Olive Sampling

In total, nine olive cultivars were selected for this work; they were grown in groves located in three different Italian regions: Sardinia, Abruzzo and Apulia.

The Sardinian cultivars “Sivigliana da olio”, Semidana and “Corsicana da olio” have been chosen for their suitability to be processed as table olives (Sivigliana da olio), for their resistance to some common diseases and high productivity (Corsicana da olio) and for the increasing interest of growers to introduce it in all Sardinian orchards (Semidana) [45,46]. Specialized non-irrigated orchards were located in the province of Sassari (Sardinia, Italy); olives of Sivigliana and Semidana cultivars were hand harvested in Ittiri (40°37′15.7″ NL 8°32′20.7″ EL), while samples of Corsicana were picked in Usini (40°39′39.0″ NL 8°31′32.7″ EL).

With regard to Abruzzo (Central Italy), the varieties Dritta, Tortiglione and Gentile dell’Aquila were selected as the most representative of the provinces of Pescara, Teramo and L’Aquila, respectively, also in consideration of their specific adaptation to the local pedoclimatic conditions correlated with peculiar agronomic and biochemical traits [47,48]. Dritta and Tortiglione were grown in orchards located in Notaresco, in the province of Teramo (42°38′54.6″ NL, 13°52′57.4″ EL), while Gentile dell’Aquila was grown in orchards from Vittorito, in the province of L’Aquila (42°08′16.6″ NL, 13°48′37.3″ EL).

In the Apulia region (Southern Italy), in addition to some well-known and notable cultivars for olive oil extraction, a great number of other varieties and/or landraces are present [3]. Among these, three have been considered in this study, specifically the varieties Bambina, Oliva Rossa and Cima di Melfi, for which few reports are currently available in the literature [49,50]. The Apulian genotype Bambina was cultivated in the orchard located in Gravina di Puglia (40°49′0″ NL, 16°25′0″ EL), while genotypes Cima di Melfi and Oliva Rossa were grown in the orchard of Putignano (40°51′0″ NL, 17°7′0″ EL), both in the province of Bari.

For the molecular characterization, young leaves of each cultivar were collected and stored at −20 °C before use. For gene expression analysis, drupes were harvested at different developmental stages of fruit, generally from mid-October to the beginning of November: T1, yellow-green olives; T2, turning olives; and T3, almost dark olives (Table 1). In order to minimize the effects of fruit asynchronous maturation within the same tree, drupes were harvested from the external parts of the canopy of trees. After harvesting, olives were immediately frozen in liquid nitrogen and stored at −80 °C until further processing. For two of the considered time-points (T2 and T3), one part of the collected drupes was immediately used for virgin olive oil extraction and biochemical characterization. An additional timepoint at 100% ripening (T4, fully dark olives, from the end of November to the beginning of December) was considered exclusively for oil production and biochemical characterization (Table 1). For each cultivar/genotype, three biological replicates were taken. Sampling was carried out for two consecutive years.

### 2.2. Genetic Characterization

Leaf samples were collected from three plants per cultivar, lyophilized and finely pulverized before use. Total genomic DNA was extracted from 200 mg of dry tissue according to [51], checked for quantity and quality and normalized to 50 ng µL^−1^. Olive genotypes were molecularly characterized by means of 12 nuclear SSRs (Table S1) [52,53,54], chosen on the basis of their suitability and reliability proven in several studies about olive variety identification [55,56,57]. Primer pairs were synthesized by Thermo Fisher Scientific (Waltham, MA, USA) and all forward primers were labeled with one of the following dyes: 6FAM™, NED™, VIC^®^ and PET™. The amplification reactions were carried out in a final volume of 12.5 µL, using a T100 thermal cycler (Bio-Rad Laboratories, Segrate, MI, Italy) according to [9]. Two microliters of each PCR product was added to 0.5 µL of GeneScan™ 600 LIZ^®^ Size Standard (Applied Biosystem, Foster City, CA, USA) and 9.5 µL of Hi-Di Formamide (Applied Biosystem, Foster City, CA, USA), and successively separated by capillary electrophoresis using an automatic sequencer ABI PRISM 3100 Avant Genetic Analyzer (Applied Biosystems, Foster City, CA, USA). Detection, sizing and data collection were carried out by means of the GeneMapper^®^ genotyping software v.5.0 (Applied Biosystems, Foster City, CA, USA) as in [58]. To estimate the genetic distances among the considered genotypes, cluster analysis based on the Unweighted Neighbor Joining method was performed using DARWIN software v. 6.0.010 (http://darwin.cirad.fr, accessed on 26 April 2019), with 1000 bootstrap values for tree construction.

### 2.3. Oil Extraction and Characterization

Around 10 kg of drupes were collected from three trees per each sampling point and variety. Fruits were then divided into three aliquots representing the biological replicates. Oil extraction was performed within 5 h after harvesting at laboratory scale. The extraction system was made up of a semi-industrial scale hammer crusher (RETSCH GmbH 5657, Haan, Germany) working at 2850 rpm and a basket centrifuge with a bowl of 19 cm working at 2700 rpm (Marelli Motori S.p.A., Arzignano, VI, Italy) [59]. Briefly, about 1 kg of olives per sample and replicate was crushed and then the olive paste was transferred into the basket centrifuge for the oil recovery. Once extracted, the virgin olive oils (*n* = 3) were stored at 20 °C in 100 mL dark glass bottles until the analyses.

Two phenolic alcohols (i.e., hydroxyl-tyrosol and tyrosol) and the oleacein (3,4-DHPEA-EDA) were chosen as target phenolic compounds for HPLC analyses, being amongst the most abundant ones in olive oils and because of their powerful antioxidant activity and impact on oil sensorial feature [60]. These phenolic compounds were extracted by liquid–liquid extraction using a mixture of methanol/water (70/30 *v*/*v*) according to previous papers [61]. The extraction procedure was similar for both the Folin–Ciocalteu assay and for HPLC analysis, with the only difference being that in the latter case 250 μL of a 100 mg kg^−1^ solution of gallic acid as internal standard for quantification was added. Total phenolic compounds (TPC) were quantified spectrophotometrically [61] by means of a calibration curve of pure gallic acid and the results are expressed as gallic acid equivalent (GAE, mg kg^−1^). HPLC-DAD analysis was carried out as previously reported [61] using a UHPLC binary system (Dionex Ultimate 3000 RSLC, Waltham, MA, USA). The identification of hydroxy-tyrosol, tyrosol and 3,4-DHPEA-EDA was performed by comparing the peak retention times with those obtained by the injection of pure standards and/or with data in the literature [62]. The results are expressed as gallic acid equivalent (GAE, mg kg^−1^).

### 2.4. Polyphenolic Compound Gene Expression

The mesocarps of frozen olives were mechanically crushed by the use of a tissue lyser and total RNA was extracted from 100 mg of the obtained powder according to the manufacturer’s instructions of Spectrum Plant Total RNA Kit (Sigma-Aldrich, St. Louis, MO, USA). An additional step for on column genomic DNA digestion was added. RNA quantity and quality were checked by spectrophotometric measurement using a Nanodrop 2000 spectrophotometer (Thermo Fisher Scientific, Waltham, MA, USA) and by electrophoresis on 1.2% Certified Molecular Biology Agarose gel (Bio-Rad Laboratories, Segrate, MI, Italy) in 1X TBE buffer (1 M Trizma base, 1 M Boric Acid, 20 mM EDTA, pH 8.3). 

Quantitative real-time polymerase chain reactions (qRT-PCRs) were carried out on three genes involved in the polyphenolic biosynthesis, named TYRD for tyrosine/dopa decarboxylase, CuAO for copper amine oxidase, and ALDH for alcohol dehydrogenase. The used primer pairs are listed in Supplementary Material (Table S2). The reverse transcription of 700 μg RNA samples was performed with the SuperScript™ VILO™ cDNA Synthesis Kit (Thermo Fisher Scientific, Waltham, MA, USA) according to the manufacturer’s instructions. qRT-PCR reactions were carried out using the SsoAdvanced Universal SYBER^®^ Green Supermix (Bio-Rad Laboratories, Segrate, MI, Italy) and the CFX96 Touch Real-Time PCR Detection System (Bio-Rad Laboratories, Segrate, MI, Italy). Thermal cycling parameters were: initial denaturation at 95 °C for 3 min, followed by 40 cycles of 95 °C for 10 s and 60 °C for 30 s. The specificity of the amplification product per each primer pair was confirmed by evaluating the melting curve through an increase of 0.2 °C every 5 s from 65 to 95 °C. For qRT-PCR assay, each amplification reaction was run in triplicates of three biological replicates. The elongation factor 1α (EF1α, AM946404) was selected as a reference gene for normalization and the comparative Ct method (2^−ΔΔCt^ method) was used to analyze the expression levels of the selected genes [63].

### 2.5. Statistical Analysis

All values of chemical and genetic analysis are means (*n* = 3) ± standard deviation (sd). The statistical analysis was performed, for samples of each region, by one-way analysis of variance (ANOVA) with sampling time as the group factor and post-hoc Fisher’s LSD test, using the software Statistica 10.0 for Windows. Differences were considered to be significant when *p* < 0.05.

## 3. Results

### 3.1. Genetic Characterization

The chosen nuclear microsatellites successfully amplified all the olive cultivars under analysis, with identical genetic profiles among the replicates of each cultivar. With the exception of missing data for only three loci related to the markers DCA09, DCA17 and GAPU101, unique alleles for all the used SSR were assigned to each cultivar, thus allowing an accurate varietal identification (Table 2 and Figure S1).

**Figure 1:**
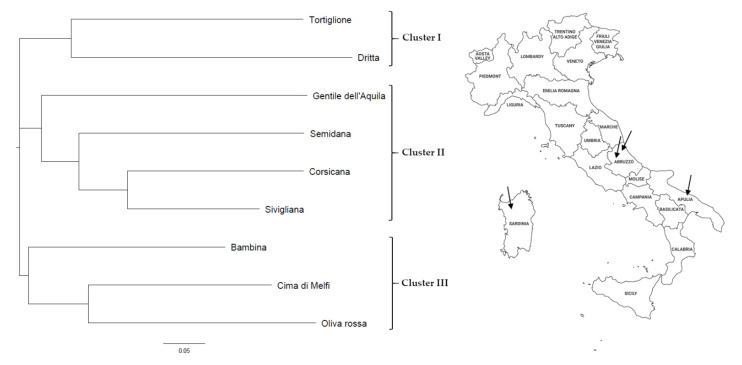
Unweighted Neighbor Joining dendrogram illustrating the clusterization of the olive cultivars under study, and a map of Italy with indication of the considered regions and the corresponding drupe sampling sites.

The Unweighted Neighbor Joining dendrogram allowed us to group the genotypes predominantly on the basis of their regional origin, except one. Indeed, as expected, three main clusters were identified (Figure 1). Cluster I included two of the three analyzed Abruzzo cultivars, i.e., Tortiglione and Dritta. The third Abruzzo cultivar, Gentile dell’Aquila, was included in Cluster II together with all the Sardinian cultivars (Semidana, Sivigliana and Corsicana). Finally, Cluster III encompassed cultivars from the Apulia region, i.e., Bambina, Cima di Melfi and Oliva Rossa.

### 3.2. Monovarietal Olive Oil Phenolic Content

**Figure 2:**
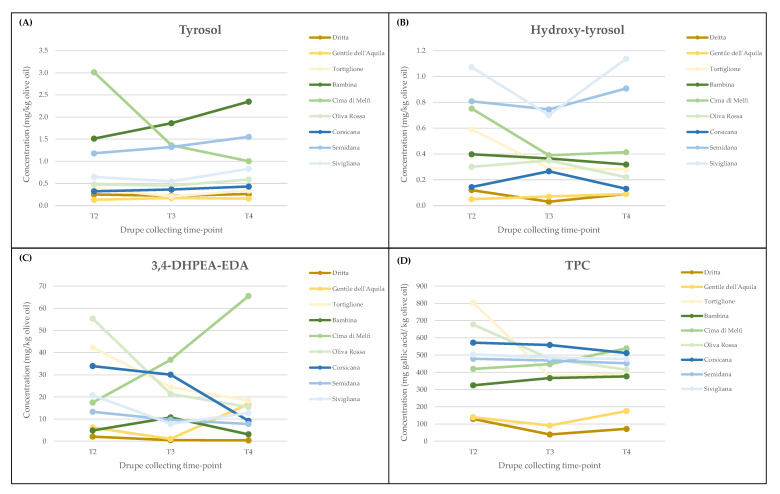
Trend of variation in some polyphenolic compound contents in VOOs of the analyzed genotypes, during three developmental stages of olive fruits (T2, T3, T4). (**A**) Tyrosol; (**B**) hydroxy-tyrosol; (**C**) oleacein (3,4-DHPEA-EDA); (**D**) total phenol content (TPC).

This research focused on three of the main active constituents of olive polyphenols, i.e., tyrosol, hydroxy-tyrosol and oleacein (3,4-DHPEA-EDA), and on the TPC of monovarietal oils. In general, the concentration and the main trend of these compounds varied among the analyzed cultivars. For example, tyrosol content was higher in cultivar Tortiglione than in Dritta and Gentile dell’Aquila with no significant changes (*p* > 0.05) during ripening, while it significantly increased in Sardinian cultivars and in two of the Apulian genotypes, i.e., Bambina and Oliva Rossa. An opposite trend was observed in cultivar Cima di Melfi, that showed a significant drop in tyrosol content at complete maturity of drupes (Table 3; Figure 2A).

Hydroxy-tyrosol concentration was the lowest among the three studied compounds, generally showing its highest amount in two time-points, that is at T2 and T4. Among all the analyzed cultivars, two Sardinian cultivars (Semidana and Sivigliana) had the highest hydroxy-tyrosol content, with values of about 0.9 and 1.2 mg kg^−1^ olive oil, respectively. In particular, in Sivigliana cultivar, this compound first decreased when drupes were in advanced stage of ripening (T3) and then significantly increased at the end of fruit maturation. An exception to this behavior was recorded for cultivars Oliva Rossa and Corsicana, whose hydroxy-tyrosol contents were slightly higher at T3 (Table 3; Figure 2B).

The concentration of oleacein generally decreased with maturity stage in all the considered cultivars, with the only exception of Cima di Melfi and Gentile dell’Aquila, that showed a more evident and significant increase in this compound in fully ripe fruits (Table 3; Figure 2C). Cultivars Dritta and Bambina were the genotypes with the lowest amount of 3,4-DHPEA-EDA in their oils. Bambina VOO was already known for its particular phenolic pattern, which shows a significant contribution of flavonoids and a lesser amount of secoiridoid derivatives [49].

Finally, changes in the total phenol content (TPC) were generally recorded in most of the cultivars. Indeed, the concentration of total phenols was almost constant during drupe maturation or slightly increasing at the last stage of fruit ripening (T4), except for the Abruzzo cultivar Tortiglione and the Apulian cultivar Oliva Rossa, that showed a drastic and significant drop in the total phenol content in comparison with the first stage of fruit ripening (T4 vs. T2) (Table 3; Figure 2D). Sardinian cultivars showed, on the other hand, a slight but significant decrease in TPC at the T4 sampling. Anyway, the oils obtained from these cultivars, together with oils derived from most of the Apulian genotypes, were the ones with the highest and most stable amount of total phenols during all stages of fruit ripening.

### 3.3. Gene Expression Analysis

**Figure 3:**
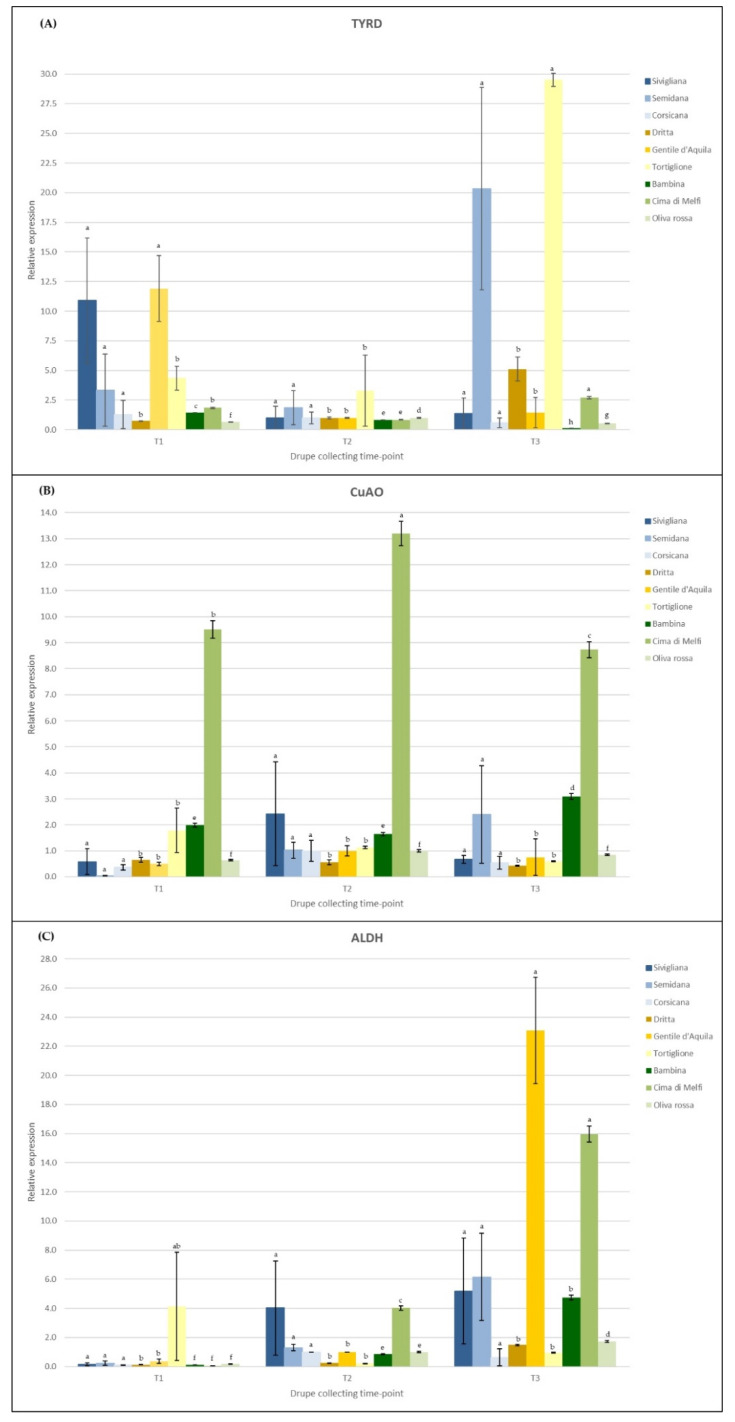
Expression profiles of the analyzed genes involved in the first step of the phenol biosynthetic pathway during fruit development (T1 = yellow-green drupes, T2 = turning drupes, T3 = almost dark drupes). The mRNA levels were determined by qRT-PCR and relatively expressed as ΔΔCt. (**A**) TYRD = tyrosine/dopa decarboxylase; (**B**) CuAO = copper amine oxidase; (**C**) ALDH = alcohol dehydrogenase. Data are means (*n* = 3) ± standard deviation. Different letters indicate significant differences according to one-way ANOVA followed by Fisher’s LSD post-hoc test (*p* = 0.05).

The expression profiles of the selected genes did not follow the same trend among the cultivars. In most genotypes, the mRNA levels of TYRD were quite low and remained almost constant as the fruit development proceeded (Figure 3A). An exception was represented by cultivars Sivigliana and Gentile dell’Aquila, in which this gene was highly expressed at the first drupe sampling (T1) and then drastically dropped at the second sampling, albeit with no significance. On the contrary, in cultivars Semidana and Tortiglione, TYRD mRNA levels remarkably increased from the second to the third sampling, thus reaching the highest values when drupes were almost dark and mature.

In some cultivars, the CuAO expression pattern did not notably change during fruit maturation, while in other genotypes, such as Semidana and Bambina, a slight increase was observed up to the third sampling time. Interestingly, the CuAO expression level was noteworthy in one of the Apulian cultivars, i.e., Cima di Melfi, not for its general trend but specifically for its abundance during the entire drupe maturation process, since it was extremely and significantly different (higher) in comparison to that detected in all other cultivars (Figure 3B).

In the majority of genotypes, ALDH was almost exclusively expressed during the T3 stage of fruit development, with a remarkable increase starting at the turning phase of drupes, in particular in the cases of Gentile dell’Aquila and Cima di Melfi, and with a less pronounced increment observed in the cases of Bambina, Semidana and Sivigliana. On the contrary, ALDH expression levels in the remaining cultivars were very low (Figure 3C).

## 4. Discussion

The identification of minor olive cultivars with favorable agronomic and nutraceutical features among the available and still uncharacterized Italian germplasm, and their fine characterization with different approaches, could effectively contribute to both the valorization and the spread of these cultivars worldwide. The possibility of their introduction in the olive-growing sector is reinforced by the availability of a molecular fingerprint, that can provide a crucial tool for the identification of these genotypes and the traceability of their oils. The use of microsatellite markers in this study has allowed not only a fine characterization of the considered genotypes, but also clarification of the phylogenetic relationships among them and assessment of an identification key. The Unweighted Neighbor Joining dendrogram mostly separated the genotypes according to their geographic origin as reported in many other studies about the evaluation of the genetic diversity of the Italian olive germplasm [5,64]. Surprisingly, only one of the analyzed genotypes has escaped this kind of clustering; indeed, the Gentile dell’Aquila cultivar, belonging to the autochthonous cultivars of Abruzzo region, grouped with all the Sardinian genotypes instead of with the other cultivars from Central Italy (Figure 1). The explanation of this result can be traced back to the complex migration history of the olive. In 2015, Dìez and colleagues [65] demonstrated the existence of three different gene pools (Q1, Q2 and Q3) in the Mediterranean area and the presence of a broad ‘mosaic’ group as a mixture of Q1 and Q2. The Italian cultivars considered in that research mainly belonged to the Q2 group. Anyway, the authors postulated that the wide occurrence of the ‘mosaic’ cultivars might be indicative of admixture events. Thereby, both the outcrossing nature of *O. europaea ssp. europaea* as well as the exchanges related to human migration over the centuries [64,66] could explain a certain level of genetic admixture between Gentile dell’Aquila and the Sardinian cultivars or their origin from a common wild ancestor.

Over the last decades, virgin olive oil (VOO) became the symbol of good nutritional habits. This role has been mostly linked to some features such as the high content of MUFA and of phenolic compounds, which allow us to recognize VOO as a functional food [67]. As proof of that, the European Food Safety Authority (EFSA) has permitted the declaration “Olive oil polyphenols contribute to the protection of blood lipids from oxidative stress” when olive oil contains a minimum of “5 mg hydroxy-tyrosol and its derivatives per 20 g of olive oil” [68,69]. However, VOO phenolic content not only plays an important nutritional role but also has a great impact on the organoleptic features and on the stability of the product [60]. Given the wide spectrum of features directly linked to olive phenolics, it is clear how important the understanding of their synthesis and regulation is.

Polyphenol accumulation has been reported to be a complex process considerably varying among genotypes, tissues, developmental stages and in response to different agronomic and environmental conditions [70,71,72]. In accordance with other studies, there was a component of the variability of the phenolic composition observed among the analyzed monovarietal virgin oils that mostly depended on the genotype. On average, the Apulian cultivars generally showed the highest values of the detected compounds at the complete maturation stage of drupes, followed by the Sardinian cultivars. This could also be related to the different cultivation areas and environmental temperatures of the considered groves, since the climates of Apulia and Sardinia are generally similar to each other and usually milder than that of Central Italy (Abruzzo). As reported by [73], the pathways related to olive phenol compounds could be differentially modulated on the basis of altitude and temperatures, phenols biosynthesis being prolonged in temperate areas.

Quantitative differences in phenolic compounds were highlighted both among the analyzed cultivars and among fruits of the same cultivar at different ripening stages. With regard to total phenols (TPC), a sharp decrease is generally and mainly observed between June and September, as result of the oleuropein hydrolysis during drupe maturation (high β-glucosidase activity) [74,75]. Indeed, the very early stages of drupe formation are dominated by secoiridoids (above all oleuropein), while in the last ripening stages, simple phenols and flavonoids become the major components. In our study, the drupe collecting period between October and December did not allow us to highlight such remarkable differences in total phenol content, this period being characterized by a higher stability of phenol metabolism (Table 3; Figure 2D). Thereby, with the only exception of cultivars Tortiglione and Oliva Rossa, that showed a sharp decrease from T2 to T3 stage, the variation of TPC was minimal in all cultivars during the fruit maturation period considered in this study. An intermediate behavior was registered in oils obtained by Sardinian cultivars, that showed a slight, but significant, decrease only at the overripe (T4) stage.

On the contrary, the content of single compounds was very variable among cultivars and sampling times. Secoiridoid derivatives, i.e., aglycon forms of the secoiridoid glucosides usually formed during oil extraction by the enzymatic hydrolysis of oleuropein, demethyloleuropein and ligstroside [76], were reported to be the most abundant phenols in monovarietal olive oils [72]. Among those, oleacein (3,4-DHPEA-EDA) is an important compound, structurally similar to oleocanthal and with comparable pharmacological properties [77]. In our study, the oleacein levels considerably differed among all genotypes, progressively decreasing as fruit maturation proceeded (Table 3; Figure 2C). Indeed, the higher values were found at the T2 time-point, although with remarkable differences among cultivars (in the range between 2 and 63 mg/kg). As a result of an opposite behavior to this general trend, oils obtained by fully ripe drupes (T4) of the Apulian cultivar Cima di Melfi and of the Abruzzo cultivar Gentile dell’Aquila were those with the highest oleacein concentration. As already mentioned, variation in the VOOs’ phenolic composition can be ascribable to several factors, including the technological steps of olive oil extraction, which is far from being merely a mechanical process. Indeed, starting from drupe crushing, several enzymes are set free whose activities are strongly influenced by the genotype, the maturity degree and the environmental conditions (time, temperature, atmosphere) [78,79]. Moreover, secoiridoid derivatives, such as 3,4-DHPEA-EDA, are among the most affected olive phenols during the extraction process. For example, in Tuscan olive oils, oleacein was found only a long time after milling, likely due to the activity of different esterases [80]. Thus, linearity between drupe genetic expression and VOO phenols could be difficult to see and the identification of clear markers of maturity is a complex task [81].

The content of minor phenols such as tyrosol and hydroxy-tyrosol was also evaluated in the considered genotypes, due to their relevance as bioactive molecules with beneficial properties on human health [16,82]. Hydroxy-tyrosol is a simple alcohol conjugated to form oleuropein derivatives, usually highly expressed in young olive fruits [42,43]. In accordance with other reports, the hydroxy-tyrosol amount decreased from semi-green to nearly ripe drupes (with the exception of Corsicana, Oliva Rossa and Gentile dell’Aquila), but then an interesting increase was again observed in the final developmental stage, i.e., in totally black fruits (Table 3; Figure 2B). This result is generally attributed to oleuropein catabolism rather than to new hydroxy-tyrosol biosynthesis, as a consequence of the recycling of all the valuable molecules of complex compounds and their conversion to simple phenols [42,44,83]. Moreover, the oleuropein catabolism in the mature fruit is necessary to reduce the bitter taste typically associated with this compound.

Changes in tyrosol content with ripening did not follow a clear pattern among the studied varieties. In some cases (such as Semidana and Cima di Melfi) an opposite trend with the oleacein content was found. These variations could be supposed to be linked to the genetic expression of the enzymes in the biosynthetic pathway (Figure 3). Indeed, tyrosol could be initially synthesized—and mostly found—in free form, while subsequently it is used as a precursor for oleacein production (Table 3; Figure 2A). However, as already reported [81], finding a straightforward relationship between gene expression and phenotype is very difficult, as the interaction with agronomic and pedoclimatic conditions should be also taken into account. Moreover, the content of phenolic compounds depends also on the activity of other enzymes such as the olive *β*-glucosidases (not considered in this study) which strongly affect their release in the oily phase. Different patterns of tyrosol accumulation with respect to the cultivar and the maturity degree have been already observed by other authors [84].

**Figure 4:**
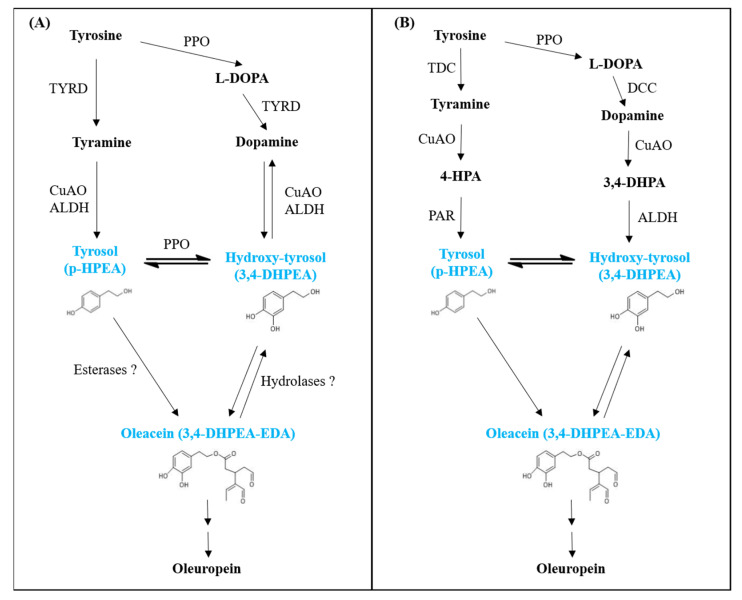
Simplified representation of the biosynthetic pathway of some important olive polyphenols. The previously identified enzymes and reactions (**A**) as well as the recently identified ones, with additional steps in the biosynthesis (**B**), are reported. Abbreviations: 3,4-DHPEA-EDA: elenolic acid linked to 3,4-dihydroxyphenyl ethanol (3,4-DHPEA); 4-HPA: 4-hydroxyphenylacetic acid; 3,4-DHPA: 3,4-dihydroxyphthalic acid; TYRD: tyrosine/DOPA decarboxylase; PPO: polyphenol oxidase; CuAO: copper amine oxidase; ALDH: alcohol dehydrogenase; DCC: DOPA decarboxylase; PAR: phenylacetaldehyde reductase. Adapted from [40,44].

When this study started, the exact polyphenol biosynthetic pathway was still not clarified in detail; therefore, among genes annotated in phenol biosynthesis, for expression analysis our choice fell on three genes (TYRD, CuAO and ALDH) on the basis of the involvement of the corresponding encoded enzymes in the first steps of this biosynthetic pathway and in relation to the compounds detected in VOOs under characterization. Tyrosine is referred to as the precursor of the secoiridoid class [85]; in particular, the biosynthesis of tyrosol and hydroxy-tyrosol has been proposed to proceed through the formation of tyramine or, otherwise, it can follow the path including L-DOPA and dopamine as intermediates (Figure 4). Initially, an enzyme similar to a tyrosine/DOPA decarboxylase (TYRD) was recognized to be responsible for the conversion of both tyrosine in tyramine and L-DOPA in dopamine [40,86] (Figure 4A). Recently, these two conversions have been clarified to take place by the action of two different enzymes, i.e., a tyrosine decarboxylase (TDC) in the first way and a DOPA decarboxylase (DCC) in the parallel path (Figure 4B) [44]. Moreover, in the previous proposed version, the subsequent steps leading to the conversion of tyramine to tyrosol and dopamine to hydroxy-tyrosol required two enzymes called CuAO and ALDH [87]. Now, the presence of other intermediates in both paths and the action of an additional enzyme, that is, a phenylacetaldehyde reductase (PAR), have been recognized (Figure 4B) [44]. The reaction of the conversion of tyrosol to hydroxy-tyrosol and vice versa seems to involve a polyphenol oxidase (PPO) [42]. Since most of the genes responsible for secoiridoid formation have been identified in a restricted number of species, some steps of the biosynthesis of oleuropein are still unclear, even though the pathway from geranyl diphosphate to secologanin has been elucidated [42,44].

The relative expression of the selected genes was monitored during three stages of fruit maturation, at T1, T2 and T3 sampling times. The expression analysis showed the general tendency of these mRNA levels to decrease or remain more or less stable in semi-green fruits and then to increase, slightly or significantly in a few cases, in semi-dark drupes (Figure 3). These results are in line with the biochemical characterization of monovarietal oils at T2 and T3 stages, which proved that at these time-points the tyrosol and hydroxy-tyrosol contents were progressively diminishing, following the same pattern as the genetic expression data. Some exceptions to this trend have been surprisingly observed for few varieties and in particular for TYRD and ALDH genes. Indeed, these two genes were highly expressed, respectively, in the Tortiglione and Semidana genotypes, and in Cima di Melfi and Gentile dell’Aquila, with a remarkable increase in their transcript levels from T2 to T3 time-points. These unexpected but interesting results, not yet reported in other olive cultivars, to our knowledge, certainly require further investigation. In order to clarify the role of TYRD and ALDH genes in these cultivars, their expression profile should be investigated at more developmental stages of drupes during more consecutive years. Protein expression experiments as well as enzymatic activity assays could also help us to understand the involvement of these genes in polyphenol metabolism of these minor cultivars. At the moment, we could hypothesize that these genotypes are effectively characterized by a high level of gene expression even towards the end of drupe maturation, contrarily to that reported in this study and, more in general, in the literature. It is also plausible that the corresponding enzymes are conveyed to other metabolisms or that some post-translational control takes place and makes these enzymes unfunctional. Exceptions apart, the general agreement of qRT-PCR results with the biochemical outcomes confirmed that hydroxy-tyrosol biosynthesis is under a transcriptional control, as also reported by other authors [40,42,43,44].

## 5. Conclusions

Unveiling the genetic potentialities of minor cultivars/genotypes could open the doors to their valorization. Besides the safeguarding of local varieties, the information provided in this work could be useful for breeding programs with selection of genotypes that, while being resilient and better adapted to specific environmental conditions, could also exploit their potentiality in terms of accumulation of bioactive compounds with important consequences on the nutritional, sensory and stability properties of VOO. With a view to an eco-sustainable and low-input agriculture, the introduction of minor cultivars may offer interesting features that, together with the extraction technology, could address the needs of a worldwide market with growing interest towards VOOs. Thus, the results and information obtained in this study are encouraging for the valorization of the still poorly explored Italian germplasm, with important both economic and scientific consequences.

## Figures and Tables

**Table 1:** Overview of the four drupe-collecting stages and type of analysis carried out in this study.

Sampling Time-Point	Drupe Status	Performed Analysis
T1	yellow-green	Gene expression
T2	turning	Gene expression + oil biochemical characterization
T3	almost dark	Gene expression + oil biochemical characterization
T4	fully dark	Oil biochemical characterization

**Table 2:** Molecular fingerprinting of the analyzed cultivars by means of 12 microsatellite markers (SA, AB and AP, respectively, indicate Sardinian, Abruzzo and Apulian varieties). Allele sizes are reported in base pair (bp).

Cultivar	Microsatellite Marker																							
	DCA03		DCA05		DCA09		DCA13		DCA15		DCA17		DCA18		GAPU45		GAPU71b		GAPU101		EMO90		EMOL	
Corsicana (SA)	232	253	198	208	162	182	120	124	246	257	109	115	177	183	181	181	127	144	192	200	186	188	190	198
Semidana (SA)	245	253	202	206	162	174	122	140	246	257	117	117	177	179	183	185	130	144	192	218	186	188	190	198
Sivigliana (SA)	243	253	206	208	162	174	120	120	246	257	113	117	179	183	181	181	127	144	198	200	188	194	190	198
Tortiglione (AB)	229	245	198	206	186	186	120	120	246	246	113	113	177	185	195	195	124	127	192	192	188	198	198	198
Dritta (AB)	229	229	208	212	186	194	120	120	246	257	113	113	177	177	181	185	127	127	190	206	186	190	192	192
Gentile dell’Aquila (AB)	243	249	206	206	162	162	120	120	246	246	-	-	179	181	183	183	144	144	200	206	188	194	198	198
Bambina (AP)	243	253	198	206	-	-	120	120	246	266	117	117	177	177	183	185	127	144	-	-	188	188	198	198
Cima di Melfi (AP)	243	253	198	206	184	184	120	120	246	246	143	143	177	181	181	185	124	144	182	182	190	190	192	198
Oliva Rossa (AP)	239	253	206	206	184	194	120	122	246	273	143	143	177	181	185	185	127	144	182	218	188	188	200	214

“-“ indicates missing data.

**Table 3:** Phenolic compounds and total phenolic content (TPC) detected in VOOs of all the analyzed cultivars from fruits at different ripening stages (T2 = turning drupes, T3 = almost dark drupes, T4 = fully dark drupes).

Region	Genotype	Drupe Collecting Time-Point	Hydroxy-Tyrosol (3,4-DHPEA)	Tyrosol (*p*-HPEA)	Oleacein (3,4-DHPEA-EDA)	TPC
Sardinia	Corsicana	T2	0.14 ± 0.12 d	0.32 ± 0.02 f	33.95 ± 0.21 a	572 ± 3 a
	Corsicana	T3	0.27 ± 0.01 d	0.36 ± 0.02 f	30.06 ± 0.17 b	558 ± 1 a
	Corsicana	T4	0.13 ± 0.01 d	0.43 ± 0.01 ef	9.20 ± 0.43 e	511 ± 1 b
	Semidana	T2	0.81 ± 0.28 bc	1.18 ± 0.17 b	13.32 ± 3.95 d	479 ± 23 de
	Semidana	T3	0.74 ± 0.19 c	1.32 ± 0.08 b	9.69 ± 0.61 e	469 ± 9 ef
	Semidana	T4	0.91 ± 0.15 abc	1.55 ± 0.12 a	7.78 ± 0.67 e	453 ± 3 f
	Sivigliana	T2	1.07 ± 0.20 ab	0.65 ± 0.10 d	20.83 ± 1.46 c	503 ± 10 bc
	Sivigliana	T3	0.70 ± 0.15 c	0.55 ± 0.04 de	7.88 ± 0.55 e	487 ± 6 cd
	Sivigliana	T4	1.14 ± 0.29 a	0.83 ± 0.04 c	12.72 ± 1.46 d	477 ± 10 de
Apulia	Bambina	T2	0.40 ± 0.06 a	1.51 ± 0.06 c	4.85 ± 0.52 b	358 ± 15 b
	Bambina	T3	0.36 ± 0.06 a	1.86 ± 0.07 b	10.75 ± 1.76 a	406 ± 10 a
	Bambina	T4	0.32 ± 0.09 a	2.35 ± 0.13 a	3.12 ± 1.83 b	392 ± 6 a
	Cima di Melfi	T2	0.75 ± 0.01 a	3.01 ± 0.01 a	17.49 ± 1.89 c	386 ± 5 c
	Cima di Melfi	T3	0.39 ± 0.06 b	1.36 ± 0.01 b	36.79 ± 2.76 b	502 ± 20 b
	Cima di Melfi	T4	0.41 ± 0.05 b	1.00 ± 0.05 c	65.52 ± 12.26 a	730 ± 11 a
	Oliva Rossa	T2	0.30 ± 0.05 ab	0.48 ± 0.02 b	55.34 ± 2.07 a	677 ± 47 a
	Oliva Rossa	T3	0.35 ± 0.05 a	0.45 ± 0.01 b	21.36 ± 3.46 b	478 ± 5 b
	Oliva Rossa	T4	0.22 ± 0.06 b	0.59 ± 0.04 a	15.46 ± 1.77 c	416 ± 19 c
Abruzzo	Dritta	T2	0.12 ± 0.02 c	0.25 ± 0.01 b	3.71 ± 0.24 de	129 ± 27 d
	Dritta	T3	0.03 ± 0.01 c	0.23 ± 0.02 b	0.50 ± 0.09 e	39 ± 2 f
	Dritta	T4	0.09 ± 0.03 c	0.26 ± 0.04 b	1.41 ± 0.08 e	71 ± 9 e
	Gentile dell’Aquila	T2	0.05 ± 0.01 c	0.17 ± 0.03 d	6.19 ± 1.37 d	123 ± 38 d
	Gentile dell’Aquila	T3	0.07 ± 0.02 c	0.13 ± 0.05 cd	1.09 ± 0.51 e	106 ± 26 de
	Gentile dell’Aquila	T4	0.09 ± 0.01 c	0.16 ± 0.02 cd	16.80 ± 1.35 c	176 ± 15 c
	Tortiglione	T2	0.59 ± 0.12 a	0.38 ± 0.07 a	42.37 ± 3.78 a	804 ± 68 a
	Tortiglione	T3	0.29 ± 0.08 b	0.21 ± 0.02 b	24.44 ± 3.72 b	377 ± 30 b
	Tortiglione	T4	0.28 ± 0.06 b	0.34 ± 0.01 a	18.34 ± 1.61 c	395 ± 6 b

Data are means (*n* = 3) ± standard deviation. Different letters for each parameter and region indicate significant differences according to one-way ANOVA followed by Fisher’s LSD post-hoc test (*p* = 0.05).

## Supplementary Materials

The following are available online at https://www.mdpi.com/article/10.3390/foods10061371/s1, Figure S1: Example of SSR electropherograms, Table S1: Microsatellite markers used in this study, Table S2: List of primers utilized for qRT-PCR analysis.
