# Supplementary material for: Bioactive Potential of Minor Italian Olive Genotypes from Apulia, Sardinia and Abruzzo
Source: Foods. 2021 Jun 14;10(6):1371. doi: 10.3390/foods10061371 (PMC8231923; doi:10.3390/foods10061371)
Supplement: Supplementary file 1 [file foods-10-01371-s001.zip › foods-1224218-SI.pdf]

Figure S1. Example of SSR electropherograms. The molecular profile of microsatellite DCA18 identified in all the analysed olive cultivars is reported. Double or single peaks correspond to heterozygous or homozygous profiles, respectively.

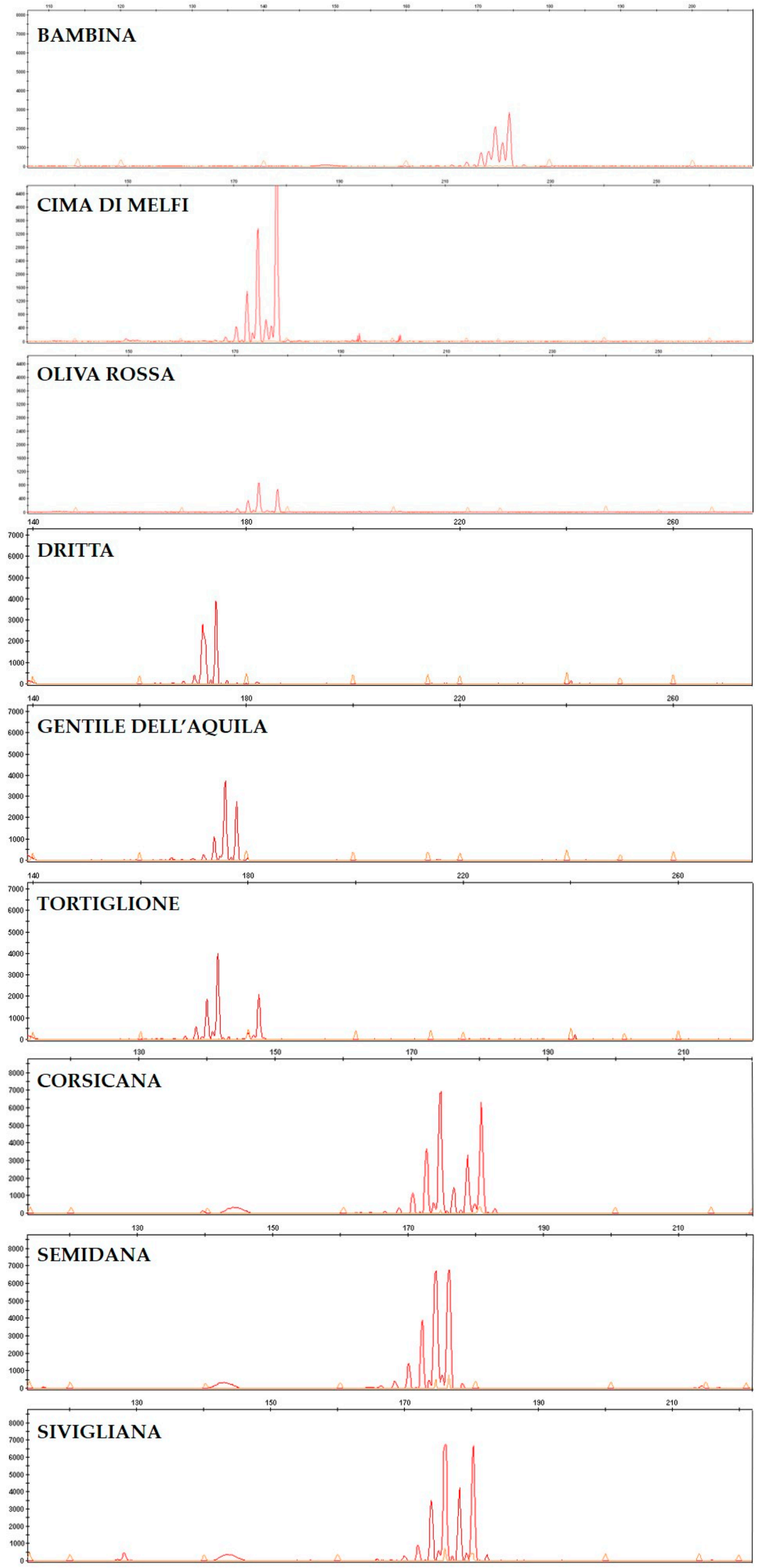

Table S1. Microsatellite markers used in this study. Locus name, repeat motif, primer sequences, annealing temperature (Ta) and allele sizing range (in base pairs, bp) are reported.

SSR locus	Repeat motif	Primer sequence (5'→3')	Ta (°C)	Allele size (bp)	Reference
DCA03	$(GA)_{19}$	CCCAAGCGGAGGTGTATATTGTTAC<br>TGCTTTTGTGTTTGAGATGTTG	50	229-257	[52]
DCA05	$(GA)_{15}$	AACAAATCCCATACGAACTGCC<br>CGTGTTGCTGTGAAGAAAATCG	50	194-214	[52]
DCA09	$(GA)_{23}$	AATCAAAGTCTTCCTTCTCATTTCG<br>GATCCTTCCAAAAGTATAACCTCTC	55	162-210	[52]
DCA13	$(CA)_{15}$	GATCAGATTAATGAAGATTTGGG<br>AACTGAACCTGTGTATCTTGCATCC	55	110-160	[52]
DCA15	$(CA)_{3}G(AC)_{14}$	GATCTTGTCTGTATATCCACAC<br>TATACCTTTTCCATCTTGACGC	50	246-273	[52]
DCA17	$(GT)_{9}(AT)_{7}AGATA(AGA)_{38}$	GATCAAATTCTACCAAAAATATA<br>TAATTTTTGGCACGTAGTATTGG	50	107-217	[52]
DCA18	$(CA)_{4}$ $CT(CA)_{3}(GA)_{19}$	AAGAAAGAAAAAGGCAGAATTAAGC<br>GTTTTCGTCTCTCTACATAAGTGAC	50	159-201	[52]
GAPU45	$(AG)_{7}$	ATCGGGAGGGATGTGATGTA<br>CATCGCATCGCCTGTAAATA	57-60	181-195	[53]
GAPU71b	$GA(AG)_{6}(AAG)_{8}$	GATCAAAGGAAGAAGGGGATAAA<br>ACAACAAATCCGTACGCTTG	57-60	118-144	[53]
GAPU101	$(GA)_{8}(G)_{3}(AG)_{3}$	CATGAAAGGAGGGGGACATA<br>GGCACTTGTTGTGCAGATTG	57-60	182-219	[53]
EMO90	$(AC)_{10}$	CATCCGGATTTCTTGCTTTT<br>AGCGAATGTAGCTTTGCATGT	50	174-200	[54]
EMOL	$(GA)_{12}$	CTTTCCAATATGGGC<br>ATGGCACTTTACGGAAAAA	50	190-214	[54]

Table S2. List of primers utilized for qRT-PCR analysis.

Gene	Accession number	Primer sequence (5'→3')	Amplicon size (bp)	Reference
OeTYRD	JX266195	TGGATCTGACCAAACCCACT<br>TTAACCCGAATGCAGTAGCC	110	[40]
OeCuAO	GQ851613	AAGATGGCCTTGGGAAGAAT<br>TTCTGCCAATCCTGTTCTCC	191	[40]
OeALDH	JX266197	TTTAAGTGGGGAGCTCAAATACA<br>GATGCTTCAGATATTCCCATGC	200	[40]
OeEF1α	AM946404	ACCACTGGTGGTTTTGAAGC<br>GAAACCAGAGATGGGGACAA	234	[40]
